# Sponge-derived alkaloid AP-7 as a sensitizer to cisplatin in the treatment of multidrug-resistant NSCLC via Chk1-dependent mechanisms

**DOI:** 10.3389/fphar.2024.1423684

**Published:** 2024-07-09

**Authors:** Li Guan, Ya-Hui Liao, Meng-Xue Cao, Li-Yun Liu, Hai-Tao Xue, Hong-Rui Zhu, Chang-Hao Bian, Fan Yang, Hou-Wen Lin, Hong-Ze Liao, Fan Sun

**Affiliations:** ^1^ Research Center for Marine Drugs, State Key Laboratory of Oncogenes and Related Genes, Department of Pharmacy, Renji Hospital, School of Medicine, Shanghai JiaoTong University, Shanghai, China; ^2^ Department of Pharmacy, Huangpu Branch, Shanghai Ninth People’s Hospital, Shanghai Jiao Tong University School of Medicine, Shanghai, China

**Keywords:** non-small cell lung cancer, multidrug resistance, aaptamine-7, Chk1, DNA damage

## Abstract

Multidrug resistance is a substantial obstacle in treating non-small cell lung cancer (NSCLC) with therapies like cisplatin (DDP)-based adjuvant chemotherapy and EGFR-tyrosine kinase inhibitors (TKIs). Aaptamine-7 (AP-7), a benzonaphthyridine alkaloid extracted from *Aaptos aaptos* sponge, has been shown to exhibit a broad spectrum of anti-tumor activity. However, the anti-cancer activity of AP-7 in combination with DDP and its molecular mechanisms in multidrug-resistant NSCLC are not yet clear. Our research indicates that AP-7 bolsters the growth inhibition activity of DDP on multidrug-resistant NSCLC cells. AP-7 notably disrupts DDP-induced cell cycle arrest and amplifies DDP-induced DNA damage effects in these cells. Furthermore, the combination of AP-7 and DDP downregulates Chk1 activation, interrupts the DNA damage repair-dependent Chk1/CDK1 pathway, and helps to overcome drug resistance and boost apoptosis in multidrug-resistant NSCLC cells and a gefitinib-resistant xenograft mice model. In summary, AP-7 appears to enhance DDP-induced DNA damage by impeding the Chk1 signaling pathway in multidrug-resistant NSCLC, thereby augmenting growth inhibition, both *in vitro* and *in vivo*. These results indicate the potential use of AP-7 as a DDP sensitizer in the treatment of multidrug-resistant NSCLC.

## 1 Introduction

Lung cancer is one of the most common malignancies globally and responsible for nearly 18% of cancer-related deaths, holding its position as the leading cause of cancer death (Sung et al., 2021; Wu et al., 2021; Siegel et al., 2023). Non-small cell lung cancer (NSCLC) constitutes 85% of all lung cancer cases, with an average 5-year survival rate of only 10%–15% (Mei et al., 2017). The clinical management of NSCLC involves a range of treatment options, including surgery, radiotherapy, chemotherapy, and targeted therapy. However, due to early metastasis, many patients are ineligible for surgical interventions (Skribek et al., 2022). Cisplatin (DDP)-based adjuvant chemotherapy, a regimen that inflicts DNA damage, has emerged as a primary treatment strategy, significantly enhancing the 5-year overall survival by 5.4% in absolute terms (Chen et al., 2014; Nikolos et al., 2018; Ramkumar et al., 2021). Furthermore, targeted therapies, such as the first-generation epidermal growth factor receptor (EGFR)-tyrosine kinase inhibitors (TKIs) like gefitinib, are standard treatment for advanced EGFR mutant NSCLC (Mei et al., 2017). Despite their initial effectiveness, the clinical success of DDP and EGFR-TKIs in NSCLC treatment is hampered by the development of acquired resistance in tumor cells (Mei et al., 2017; Liu M. et al., 2021).

Resistance mechanisms to drug therapy, particularly those involving DNA damage repair leading to apoptosis, have been extensively researched (Liu et al., 2017; Liu M. et al., 2021; Kryczka et al., 2021; Ali et al., 2022). Following DNA damage therapy, the ATM (ataxia telangiectasia mutated)/ATR (ataxia telangiectasia and Rad3-related) and downstream substrates, such as checkpoint kinase (Chk)1 and p53, are activated. These activated proteins directly phosphorylate the Cdc25 family members, preventing Cdc25 from dephosphorylating CDK1. This leads to cell-cycle arrest and DNA damage repair in tumor cells, ultimately reducing the efficacy of therapeutic drugs that induce apoptosis (Kotsantis et al., 2018; Duan et al., 2019; Xu et al., 2021). Inhibition of DNA repair pathways can prevent cancer cells from repairing DNA damage induced by treatments like chemotherapy or radiation, increasing cell death and improving treatment outcomes. Targeting the inhibition of Chk1 is a potential strategy for mitigating multidrug resistance (MDR) in NSCLC (Luo and Leverson, 2005; Gadhikar et al., 2013; Itamochi et al., 2014). Previous preclinical and clinical studies have demonstrated the efficacy of inhibiting Chk1 signaling, utilized either alone or in combination with multiple genotoxic chemotherapies, in reducing DNA damage repair and alleviating drug resistance in several cancer models (NCT02264678, NCT01139775, NCT02589522, NCT02797964).

Metabolites derived from marine sponges have gained significant attention as a valuable source for lead compounds (Ru et al., 2022). Among these metabolites, alkaloids have been identified and evaluated for their potential multiple anticancer activities, including chronic myeloid leukemia, breast cancer, liver cancer, and colon cancer (Jin et al., 2011a; Yu et al., 2014a; Zovko et al., 2014; Gong et al., 2020a; Liu B. et al., 2021). In our study, we isolated Compound 7 (named as Aaptamine-7 (AP-7) in this study), one of nine known aaptamine-type alkaloids, from the marine sponge *Aaptos aaptos* collected off the Xisha Islands in the South China Sea (Yu et al., 2014b). Previous reports have indicated that AP-7 exhibits promising antitumor, antifungal, and anti-HIV activities. For example, we demonstrated that AP-7 exhibits excellent anti-cancer properties in NSCLC cells by arresting the cell cycle and inducing apoptosis. However, the anti-cancer activity of AP-7 in multidrug-resistant NSCLC cells and the underlying molecular mechanisms have not been investigated.

In this study, we aimed to explore the potential of AP-7 in enhancing the efficacy of DDP-mediated cell death in multiple drug-resistant NSCLC cells, including those resistant to DDP and EGFR-TKIs. Our experimental findings revealed that AP-7 effectively inhibited the ATR-Chk1 signaling pathway, thereby reducing DNA damage repair, promoting DNA damage accumulation, and inducing apoptosis. Notably, AP-7 demonstrated success in restoring the sensitivity of MDR cells to DDP both *in vitro* and *in vivo.* These findings suggest that AP-7 may serve as a promising lead compound for the development of anti-NSCLC drugs.

## 2 Materials and methods

### 2.1 Chemicals and materials

AP-7 was isolated from *Aaptos aaptos* by our research group and can be obtained by contacting the authors. AP-7 was dissolved in dimethyl sulfoxide (DMSO) and stored at −20°C. Working solution of AP-7 was diluted in fresh medium. Primary antibodies of Nanog, CD44, ALDH1, CDK1, p-Cdc2, Chk1, p-Chk1, γ-H_2_AX, ABCG2, β-actin and GAPDH were purchased from Cell Signaling Technology and Abcam (Supplementary Table S6).

### 2.2 Cell cultures

The human NSCLC cell lines A549, A549/cisplatin resistance (A549/DDP), and PC9 were obtained from the Shanghai Institute of Cell Biology, Chinese Academy of Sciences (Shanghai, China). The PC9-gefitinib resistance cell line (PC9-GR) was established by our laboratory using its parental cells, as previously described (Liu et al., 2020). NSCLC cells PC9-Nanog^+^ cells were derived from PC9 cells and harbored plasmid vectors encoding Nanog cDNA as previously described by our laboratory (Liu et al., 2020). Cells were cultured in RPMI-1640 (PC9, A549, A549/DDP, PC9-Nanog^+^), DMEM (PC9-GR) (Gibco, United States) medium supplemented with 10% fetal bovine serum (FBS) (Gibco, United States), 100 U/mL penicillin, and 100 μg/mL streptomycin. All these cells were maintained in a humidified atmosphere with 5% CO_2_ at 37°C.

### 2.3 Immunofluorescence assay

The cells were fixed, permeabilized and additional blocked. Cells were sequentially incubated with indicated primary antibodies overnight at 4°C, and then incubated with secondary antibody at room temperature (RT) for 1 h. The nuclei were counterstained with DAPI for 5 min. Finally, the fluorescence images were captured by laser confocal microscopy (Leica SP8). Then, cells were resuspended in 200 µL of PBS and measured by Attune NxT flow cytometer (Thermo Fisher Scientific, Eugene, Oregon, United States).

### 2.4 Cell viability assay

Cell viability was determined by CCK-8 assay. Cells were seeded in 96-well plates at a density of 3 × 10^3^ cells/well and grown for 24 h to adhere at 37°C. Then, different concentrations of therapeutic drugs or compounds were added to 96-well plates for an indicated time. Then CCK-8 reagent was added to each well, and the cells were further incubated for 0.5–4 h at 37°C. Afterward, the absorbance was measured using a microplate (spectra MAX190; Molecular Devices, United States).

### 2.5 Cell clone formation assay

Cells were seeded in 6-well plates at a density of 1 × 10^3^ cells/well and grown for 24 h at 37°C. Then, different therapeutic drugs or compounds were added to 6-well plates for 48 h. The dosed medium was replaced with fresh medium for continuous culture for 8–9 days. The cells were fixed, dyed, washed, air-dried at RT and photographed.

### 2.6 Cell cycle analysis

After treatment with therapeutic drugs or compounds for 24 h, the cells were collected and washed with PBS twice and then fixed with ice-cold 70% ethanol for overnight at 4°C. PI/RNase staining buffer (BD Pharmingen, San Diego, CA, United States) was then added and incubated with cells at RT for 15 min. The cell cycles were measured and analyzed by flow cytometry.

### 2.7 Apoptosis assay by TUNEL staining

Cells were plated in six-well plates (1 × 10^5^ cells/well) and allowed to attach for 24 h. The cells were treated with AP-7 and/or DDP for 72 h. Then, the cells were harvested, washed with PBS twice, fixed and permeabilized, then, tumor were stained with TUNEL and imaged by fluorescence microscope (Nikon, Japan).

### 2.8 *In vitro* kinase assays

Firstly, enzyme, substrate, ATP and inhibitors AP-7 or the Chk1 inhibitor AZD7762 were diluted in Kinase Buffer, respectively using the CHK1 Kinase Assay Kit (Cat. V1941, Promega Corporation) according to the manufacturer’s protocol. Then, 1 μL of inhibitor or (5% DMSO), 2 μL of enzyme, 2 μL of substrate/ATP mix were added to the wells of 384 low volume plate, and incubated at RT for 1 h. 5 μL of ADP‐Glo™ Reagent in the ADP-GlotTM Assay Kit (Cat. V9101, Promega Corporation) was added to plate and incubated at RT for 40 min. Finally, 10 μL of Kinase Detection Reagent was added and incubate at RT for 30 min, and analyzed by microplate.

### 2.9 Western blotting

For the extraction of total protein, the cells were harvested, lysed with RIPA buffer (Beyotime Biotechnology, Beijing, China), and centrifuged for 20 min (12,500 *g*, 4°C). The supernatant was collected, and the total proteins were quantified by BCA protein assay kit (Beyotime Biotechnology). Equivalent amounts of protein were separated by sodium dodecyl sulfate-polyacrylamide gel electrophoresis (SDS-PAGE) and then transferred to polyvinylidene fluoride (PVDF) membrane. The membranes were blocked with blocking solution for 1 h at RT and then incubated with specific primary antibody at 4°C overnight. Next day, the membranes were repeatedly washed and then incubated with HRP-conjugated secondary antibody for 2 h at RT. After washing for three times, the protein bands were detected and profiled by Amersham Imager 600 gel imaging system (GE Healthcare, United States).

### 2.10 Xenograft assay

Male BALB/c nude mice, aged 4∼5 weeks, were purchased from Shanghai Laboratory Animal Center (Shanghai, China). PC9 cells (1 × 10^6^) and PC9-GR cells (1 × 10^6^) were subcutaneously inoculated into the right flank of nude mice, respectively (n = 5 per group). The nude mice bearing PC9 and PC9-GR xenografts were treated with 50 mg/kg gefitinib via intraperitoneal injection for 21 days. The volume of tumors was recorded. Then the nude mice bearing PC9-GR xenografts were treated with vehicle, DDP (2 mg/kg), AP-7 (20 mg/kg), or the combination (DDP-1 mg/kg, AP-7–10 mg/kg) for 25 days. The drugs were injected intraperitoneally. All mice were euthanized after 25 days administration. Harvested tumors were imaged and weighed immediately. All the animal experiments were conducted in compliance with Animal Care and Use Committee of Ren Ji Hospital, Shanghai, China.

Then, tumors were fixed with 4% formaldehyde and stained with γ-H_2_AX, p-Chk-1, H&E, Ki67, and TUNEL respectively to analyze histological changes, proliferation and apoptosis.

### 2.11 Statistical analysis

All the statistical analysis was performed using the SPASS 17.0 (SPSS Inc., Chicago, IL, United States) and GraphPad Prism 5.0 software (GraphPad Software Inc., La Jolla, CA, United States). All data were expressed as mean ± SD of at least three experiments. Statistical significance was calculated by one-way analysis of variation (ANOVA) and Student’s *t*-test, and *p* < 0.05 was considered statistically significant.

## 3 Results

### 3.1 Multiple resistant models of different subtypes in NSCLC constructed

Our previous study successfully generated two drug-resistant cell strains derived from NSCLC cells, which provides valuable tools for screening active compounds capable of overcoming drug resistance.

**FIGURE 1:**
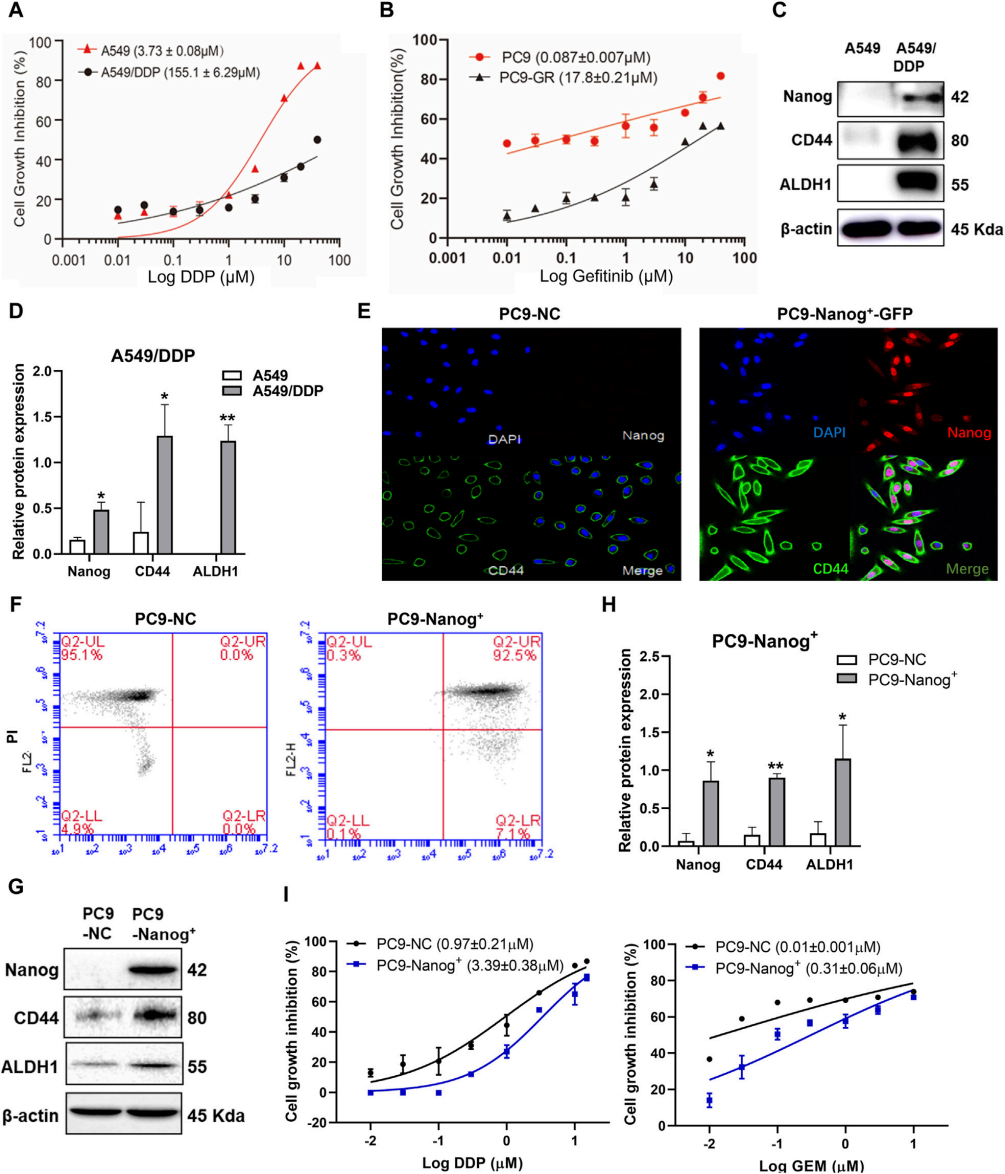
The NSCLC resistant cells exhibited MDR and cancer stem like properties. Cell viability of **(A)** A549/DDP cells and **(B)** PC9-GR cells was assessed using the CCK-8 assay after treatment with DDP or gefitinib. **(C and D)** The expression levels of Nanog, CD44 and ALDH1 in A549/DDP cells were examined by Western blot. Quantitative analysis of relative protein expression was shown as Mean ± SD (n ≥ 3). Immunofluorescence staining was performed to evaluate the colocalization of Nanog and CD44 in PC9-NC and PC9-Nanog^+^ cells **(E)**. The expression of stem cell markers was analyzed by **(F)** flow cytometry and **(G and H)** Western blot. **(I)** The CCK-8 assay was employed to assess the cell viability of PC9-Nanog^+^ cells following treatment with chemotherapeutic agents DDP and GEM. *p < 0.05, **p < 0.01, compared with the control group.

The results, as depicted in Figure 1A and Supplementary Table S1, demonstrated that A549/DDP cells exhibited significant multidrug resistance compared to the parental cells, with a resistance index of 41.55 for DDP. Additionally, the viability of PC9-GR cells in response to gefitinib, DDP, and gemcitabine (GEM) (Figure 1B; Supplementary Table S2), confirmed the acquired resistance of PC9-GR cells compared to the parental cells.

Our previous study also revealed the crucial role of the transcription factors Nanog in maintaining the CSC-like properties of NSCLC, which is closely associated with MDR (Liu et al., 2020). The overexpression of CSC markers Nanog, CD44, and ALDH1 was confirmed in A549/DDP (Figures 1C,D) and PC9-GR cells (Liu et al., 2020). To further investigate the impact of Nanog on multidrug resistance, we utilized PC9-Nanog^+^ cells, which were genetically modified to overexpress Nanog via plasmid vectors harboring Nanog cDNA (Figures 1E,F). Moreover, significantly increased expression levels of CSC markers CD44 and ALDH1 were observed in PC9-Nanog^+^ cells compared to the control cells (Figures 1G,H).

Our findings indicate that PC9-Nanog^+^ cells exhibit reduced sensitivity to DDP and GEM compared to PC9-NC cells (Figure 1I; Supplementary Table S3). These results suggest that A549/DDP, PC9-GR, and PC9-Nanog^+^ cell lines display both multiple drug resistance and CSC-like properties, making them suitable for investigating the drug resistance mechanism in NSCLC.

### 3.2 DNA damage response observed in multidrug-resistant NSCLC cells following chemotherapy treatment

**FIGURE 2:**
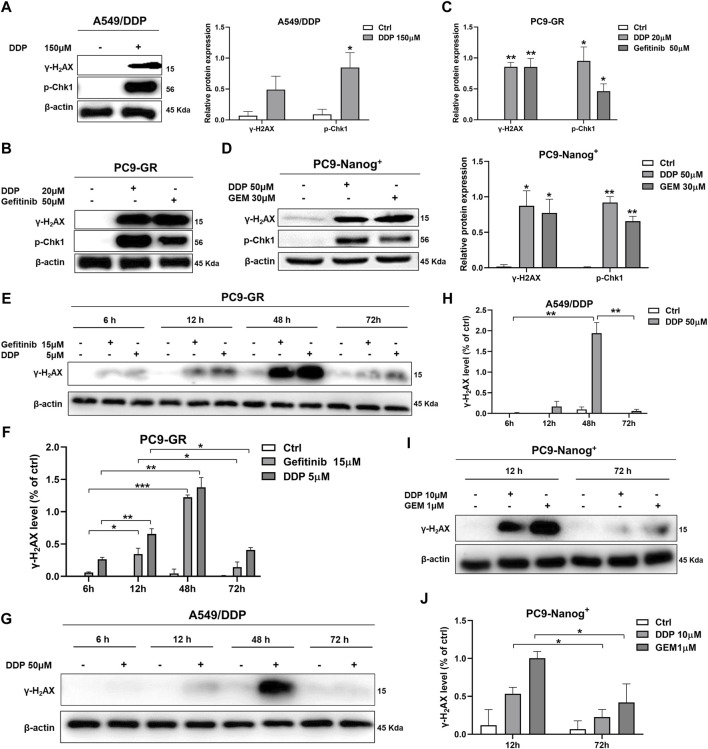
DNA damage response in multidrug-resistant NSCLC cells following treatment with chemotherapy and EGFR-TKIs. Western blot analysis was performed to detect the expression of phosphorylated histone γ-H2AX, a DNA damage regulatory protein, and activated Chk1 in **(A)** A549/DDP cells, **(B and C)** PC9-GR cells, and **(D)** PC9-Nanog^+^ cells after treatment with chemotherapy drugs and EGFR-TKIs. The changes in γ-H2AX levels in PC9-GR cells **(E and F)** and A549/DDP cells **(G and H)** were assessed at different time points (6, 12, 48, and 72 h) following chemotherapy treatment. **(I and J)** The reduction in DNA damage after 72 h of treatment with DDP or GEM compared to 12 h of treatment was also evaluated in PC9-Nanog^+^ cells by Western blot analysis. *p < 0.05, **p < 0.01, ***p < 0.001, compared to the control group.

The present study investigated the DNA damage response in multidrug-resistant NSCLC cells following drug therapy to explore the mechanisms of resistance. The results of Western blot analysis indicate that treatment with chemotherapy drugs, including DDP, GEM, and/or EGFR-TKIs gefitinib, led to increased expression of phosphorylated histone γ-H2AX and activation of Chk1 in A549/DDP cells (Figure 2A; Supplementary Figure S1), PC9-GR cells (Figures 2B,C), and PC9-Nanog^+^ cells (Figure 2D) after 12 h of exposure to these agents.

Furthermore, we examined the effects of different treatment durations of DDP or GEM on the expression of γ-H2AX in multidrug-resistant NSCLC cells. Our results, as shown in Figures 2E,F, revealed that the levels of γ-H2AX were upregulated in PC9-GR cells after 6–48 h of treatment with DDP or gefitinib, but significantly downregulated after 72 h. Similar observations were also made in A549/DDP cells, as depicted in Figures 2G,H. These findings suggest that DNA damage is repaired at 72 h of DDP or gefitinib treatment in PC9-GR cells. Moreover, in PC9-Nanog^+^ cells, the levels of DNA damage were significantly reduced after 72 h of treatment with DDP and/or GEM compared to the levels observed at 12 h of treatment (Figures 2I,J). This finding implicate that the repair of DNA damage mechanisms could play a role in the resistance of multidrug-resistant NSCLC cells to chemotherapy or EGFR-TKIs.

### 3.3 AP-7 enhances DDP-induced DNA damage and growth inhibitory effects in multidrug-resistant NSCLC cells

**FIGURE 3:**
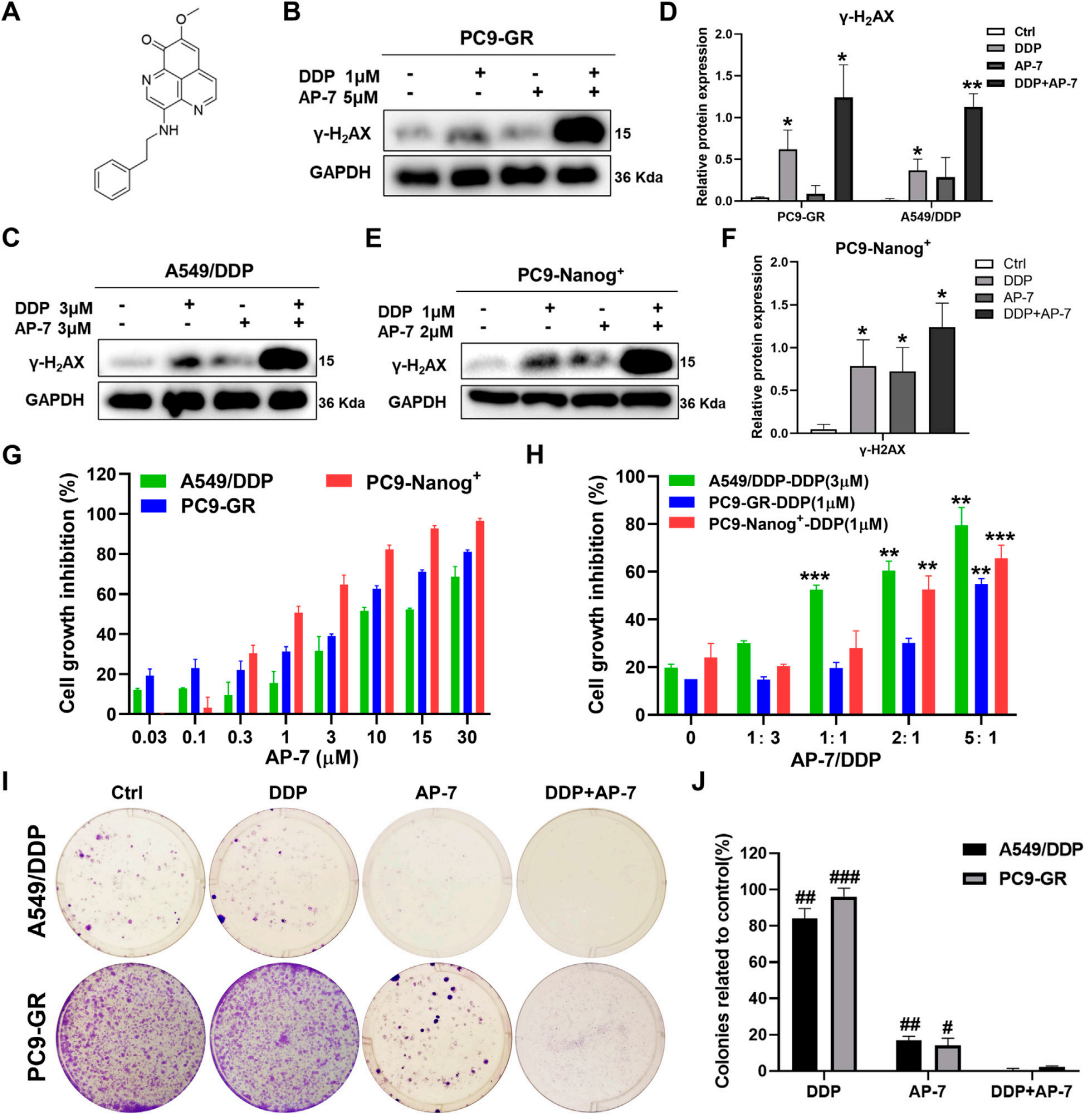
AP-7 enhances cisplatin-induced DNA damage and growth inhibitory effects in multidrug-resistant NSCLC cells. **(A)** Chemical structures of AP-7. The expression of γ-H2AX was detected by western blot in **(B–D)** PC9-GR, A549/DDP, and **(E,F)** PC9-Nanog^+^ cells after incubated with AP-7 and/or DDP for 24 h. GAPDH was used as a loading control. *p < 0.05, **p < 0.01, compared to the ctrl group. **(G)** Cell viability of A549/DDP, PC9-GR, and PC9-Nanog^+^ cells were assessed using the CCK-8 assay after treatment with AP-7. **(H)** Cell viability of A549/DDP, PC9-GR, and PC9-Nanog^+^ cells were assessed after treatment with different ratio of AP-7 and DDP. **p < 0.01, ***p < 0.001, compared to the AP-7-/DDP+ (AP-7/DDP = 0) group. **(I,J)** AP-7 inhibits the formation of A549/DDP and PC9-GR colonies. #*p* < 0.05, ##*p* < 0.01, ###*p* < 0.001, compared with DDP+AP-7 group.

It is crucial to identify drugs that can inhibit DNA damage repair and enhance DNA damage in multidrug-resistant NSCLC cells. AP-7 (Figure 3A), an apaptamine-type alkaloid compound isolated from the marine sponge *Aaptos aaptos*, has been shown to exhibit cytotoxic effect on various types of tumor cells (Yu et al., 2014b). In our study, we aimed to investigate the efficacy of AP-7 in combination with DDP in multidrug-resistant NSCLC cells.

Our findings revealed that AP-7 exhibited a stronger inhibitory effect on multidrug-resistant NSCLC cells (Figure 3G; Supplementary Table S4). The half maximal inhibitory concentration (IC_50_) of AP-7 on A549/DDP, PC9-GR and PC9-Nanog^+^ cells was 10.35 ± 0.82 μM, 3.32 ± 0.56 μM and 1.17 ± 0.19 μM, respectively. Additionally, we assessed the enhanced inhibitory effects of optimally combination ratio of AP-7 and DDP on multidrug-resistant NSCLC cells using CCK-8 assay. As shown in Figure 3H, the growth of A549/DDP, PC9-GR, and PC9-Nanog^+^ cells were inhibited by about half when the ratio of AP-7 and DDP was 1, 5, and 2, respectively, which showed significantly higher cytotoxicity than DDP alone. And our results demonstrated that the combination of AP-7 and DDP significantly increased DNA damage in multidrug-resistant NSCLC cells compared to the treatment of DDP alone (Figures 3B–F). Moreover, the combined treatment significantly inhibited clone formation of A549/DDP, PC9-GR, and PC9-Nanog^+^ cells (Figures 3I,J; Supplementary Figure S2). This finding provide evidences to support the synergistic effect of the combination of AP-7 and DDP in combating multidrug-resistant NSCLC cells.

### 3.4 AP-7 counteracts the DDP-induced cell cycle arrest and enhances apoptosis in multidrug-resistant NSCLC cells

The DNA damage response following drug therapy involves sensing the damage, signalling its presence to the cell, and recruiting downstream effector proteins. These effectors can induce cell cycle arrest, repair the DNA damage, or activate cell death. Therefore, we further investigated the effects of the combination of AP-7 and DDP to cell cycle arrest and cell apoptosis in multidrug-resistant NSCLC cells.

**FIGURE 4:**
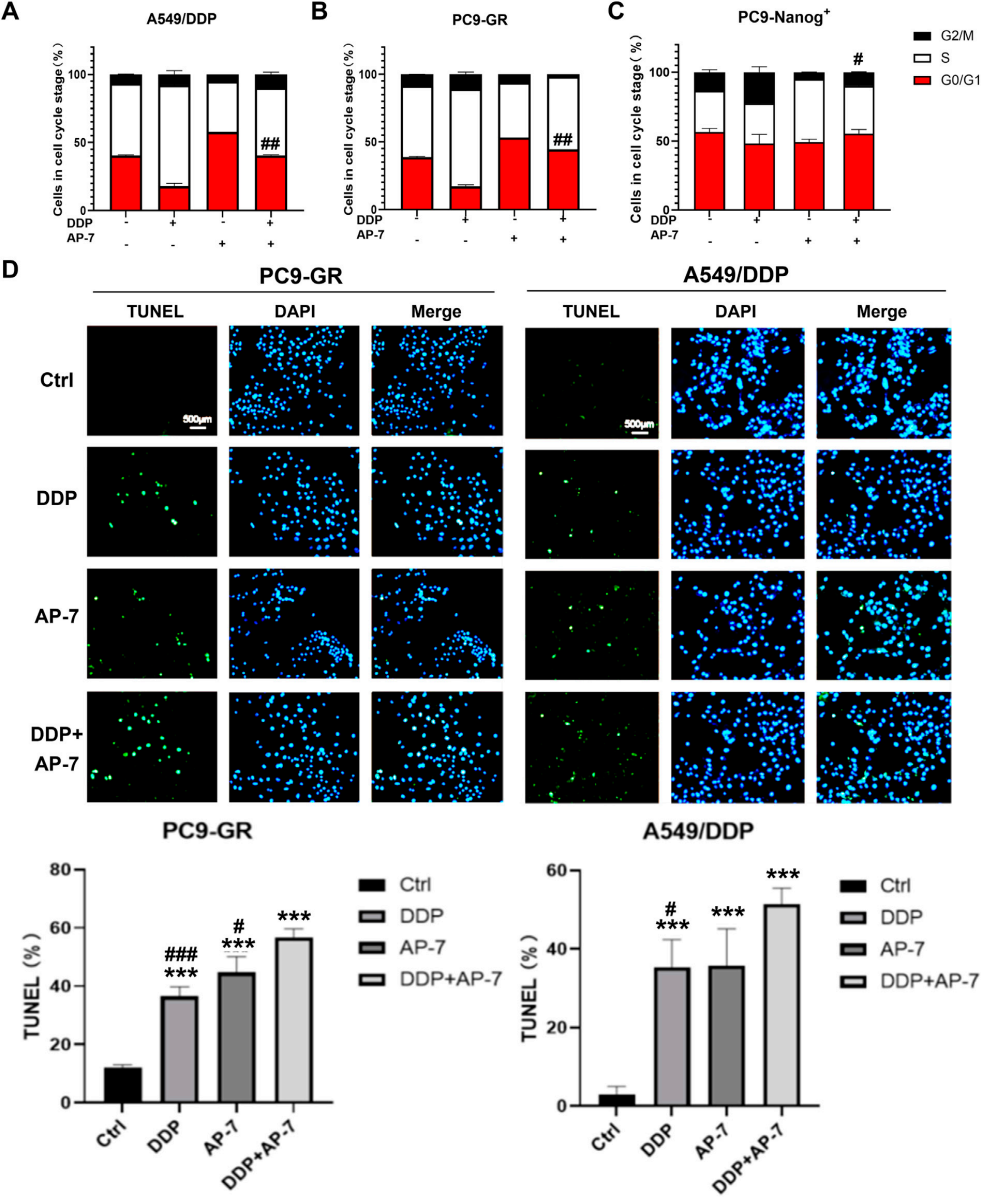
The effect of AP-7 on countering DDP-induced cell cycle arrest and enhancing apoptosis in multidrug-resistant NSCLC cells. Cell cycle distribution of **(A)** A549/DDP, **(B)** PC9-GR and **(C)** PC9-Nanog^+^ cells after treatment with AP-7 and DDP was tested by flow cytometric assay. #*p* < 0.05, ##*p* < 0.01, compared to the DDP treatment group. The mean percentage of cell cycle distribution was calculated and presented as mean ± SD from three independent experiments. **(D)** PC9-GR and A549/DDP were treated with DDP and AP-7 for 72 h, respectively, and the apoptosis rate was determined using the TUNEL assay. ***p < 0.001, compared to the control group. #*p* < 0.05, ###*p* < 0.001, compared with DDP+AP-7 group.

As shown in Figures 4A,B and Supplementary Figure S3A and S3B, DDP primarily induced cell cycle arrest at S phase, while AP-7 predominantly induced cell cycle arrest at G0/G1 phase in PC9-GR and A549/DDP cells, respectively, compared to the DDP treatment group. Furthermore, AP-7 was able to counteract DDP-induced S-phase arrest. In PC9-Nanog^+^ cells, the increased cell cycle distribution of G2/M phase induced by DDP was downregulated after treatment with AP-7 and DDP (Figure 4C; Supplementary Figure S3C).

Additionally, we investigated cell apoptosis in PC9-GR and A549/DDP cells after the combined administration of AP-7 and DDP. As depicted in Figure 4D and Supplementary Figure S4, the apoptosis rate of the combined treatment in PC9-GR and A549/DDP cells was significantly higher than that of DDP alone or AP-7 alone, suggesting that the combined use of AP-7 and DDP significantly increased the apoptosis of multidrug-resistant NSCLC cells. These results clearly demonstrate that AP-7 enhances the sensitivity of NSCLC cells to chemotherapeutic agents by attenuating the cell cycle arrest caused by DNA damage, thereby facilitating the elimination of multidrug-resistant cancer cells.

### 3.5 Combination treatment of AP-7 and DDP activates DNA damage-related signaling pathway in multidrug-resistant NSCLC cells

**FIGURE 5:**
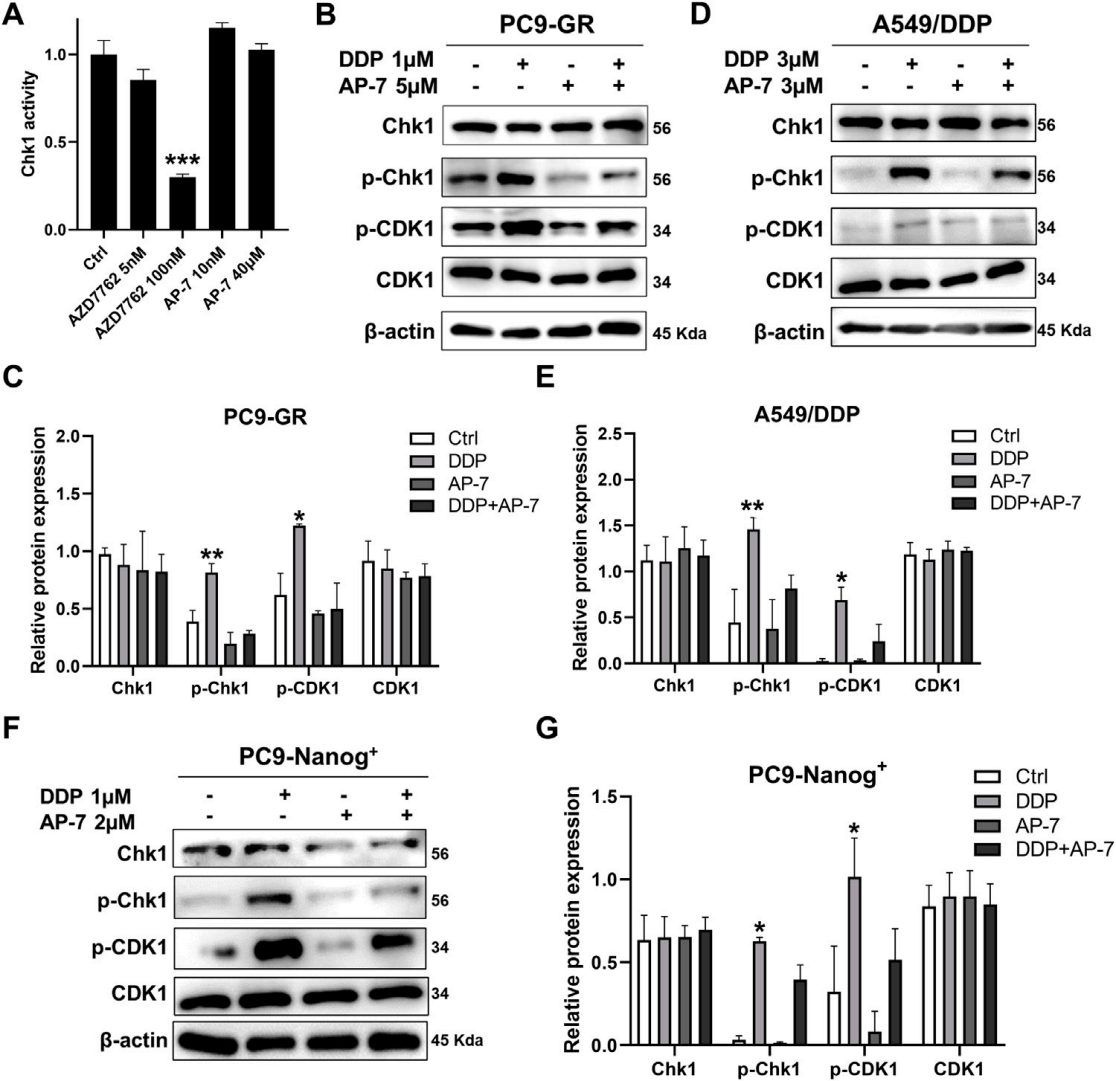
Combination treatment of AP-7 and DDP activates DNA damage-related signaling pathway in multidrug-resistant NSCLC cells. **(A)** Chk1 kinase activity was assessed by treating it with a Chk1 inhibitor and AP-7. **(B,C)** PC9-GR, **(D,E)** A549/DDP and **(F,G)** PC9-Nanog^+^ cells were treated with AP-7 and DDP for 24 h, western blot was conducted to detect the protein levels of the Chk1/CDK1 pathway. ***p < 0.001, compared to the control group. *p < 0.05, **p < 0.01, compared with DDP+AP-7 group.

To gain further insight into the molecular mechanism underlying the ability of AP-7 in combination with DDP to induce DNA damage in multidrug resistant NSCLC cells, we performed an ADP-Glo assay and conducted Western blot analysis to investigate the impact of combination treatment on DNA damage response pathways. As depicted in Figure 5A, Chk1 kinase activity was assessed, and it was observed that AP-7 had no significant effects on Chk1 kinase activity when compared with the Chk1 inhibitor AZD7762.

In Figures 5B,C, it is demonstrated that AP-7 effectively inhibits the DDP-triggered the Chk1/CDK1 pathway in PC9-GR cells. Similar results were observed in A549/DDP cells (Figures 5D,E) and PC9-Nanog^+^ cells (Figures 5F,G). In addition, the expression of drug-resistant protein ABCG2 in PC9-GR and A549/DDP cells was effectively inhibited by AP-7 and further suppressed after AP-7/DDP treatment (Supplementary Figure S5). Collectively, our results suggest that AP-7 can effectively inhibit the Chk1/CDK1 DNA damage response pathway, thereby reducing DNA damage repair, and enhance the cytotoxicity of DDP in multidrug-resistant NSCLC cells.

### 3.6 Combination treatment of AP-7 and DDP inhibits gefitinib-resistant tumor growth *in vivo*

**FIGURE 6:**
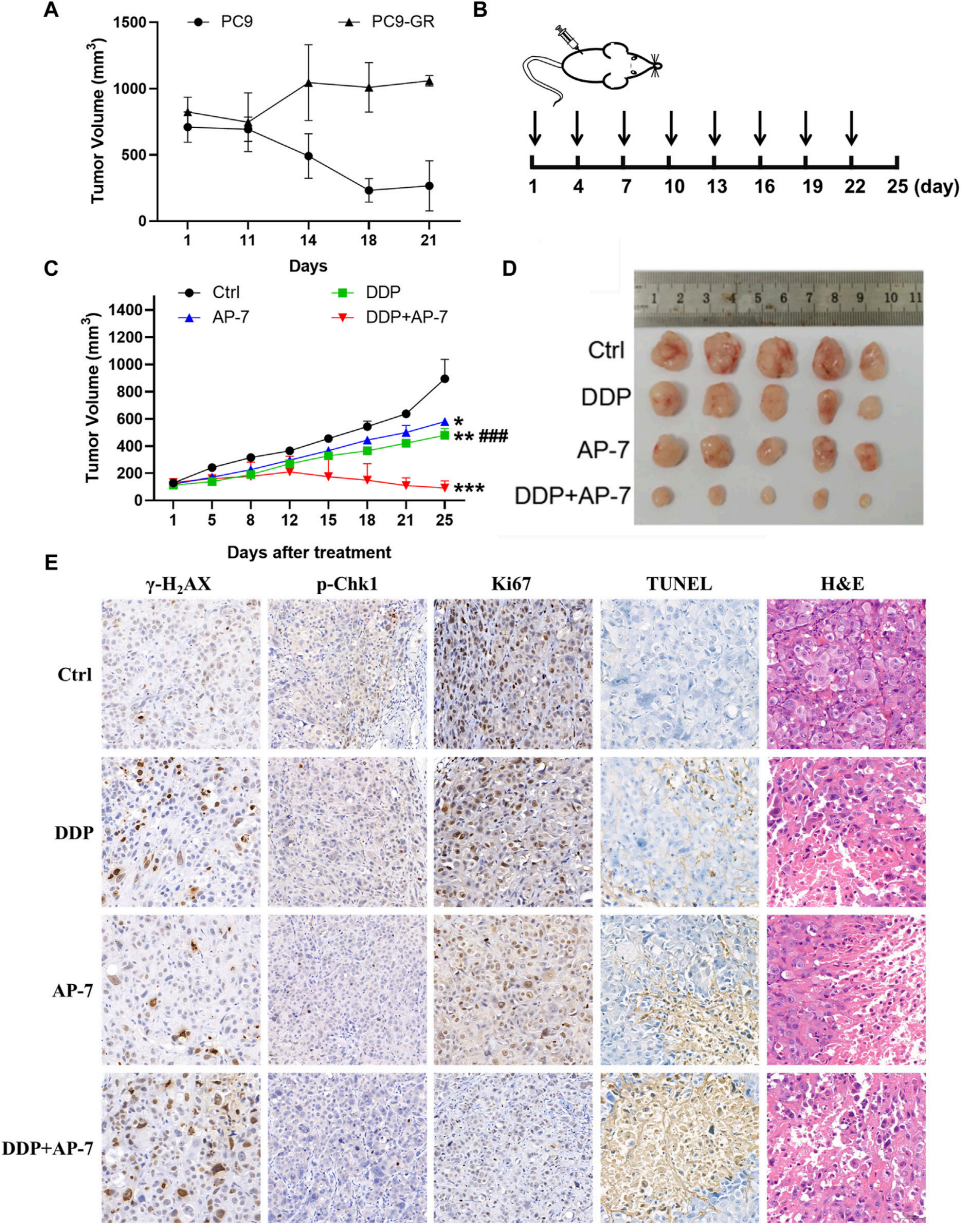
AP-7 combined cisplatin inhibits the growth of gefitinib-resistant tumor *in vivo*. **(A)** Nude mice were treated with intragastric administration of 50 mg/kg gefitinib and divided into PC9 group and PC9-GR group, the volume of tumors was recorded. **(B)** Schedule of treatment administration is shown (n = 5, black arrows indicated the time of administration). **(C)** Tumor volumes of different treatment groups were measured by caliper (mean ± SD). **(D)** Photographs of tumor from each treatment group. **(E)** Immunohistochemistry results of excised tumor tissues, including Ki67, TUNEL, H&E, γ-H_2_AX, and p-Chk1 staining (×20 magnification). ###*p* < 0.001, compared with DDP + AP-7 group. *p < 0.05, **p < 0.01, ***p < 0.001, compared to the ctrl group.

To evaluate the enhanced cytotoxicity of the combined treatment of AP-7 and DDP, we established a gefitinib-resistant xenograft tumor model in nude mice through subcutaneously inoculated with drug-resistant PC9-GR cells. After 21 days of intragastric administration of gefitinib, PC9-GR xenografts tumors continued to grow while parental PC9 xenografts tumors were significantly inhibited by gefitinib (Figure 6A). These indicated that the PC9-GR xenografts exhibited notable resistance compared to the parent PC9 xenografts, and PC9-GR xenografts could be used as a gefitinib-resistant xenograft tumor model to evaluate the treatment of AP-7 and DDP in drug-resistant NSCLC.

In accordance with the treatment schedule, the administration doses of DDP and AP-7 was 2 and 20 mg kg^−1^, respectively, whereas the combined treatment group received halved doses (Figure 6B). As shown in Figures 6C,D and Supplementary Table S5, AP-7 effectively inhibited the growth of PC9-GR tumors (*p* < 0.05, T/C ratio of 64.99% ± 0.64%), and combined with DDP significantly increased the tumor inhibitory effect of DDP (*p* < 0.001, T/C ratio of 9.04% ± 4.36%). Our results demonstrated that the combined treatment significantly inhibited the growth of the gefitinib-resistant lung cancer xenograft tumors in mice, as evidenced by the decreased tumor volume.

Immunohistochemical analysis revealed that the combined treatment group exhibited significant inhibition of Chk1 phosphorylatio compared to DDP treatment group. Furthermore, γ-H2AX staining demonstrated clearer particles and stronger DNA damage of combined treatment group compared to control and single-agent treatment groups (Figure 6E). Additionally, Ki67 staining demonstrated fewer tumor cell proliferation in the combination group, while TUNEL staining indicated a higher rate of tumor cell apoptosis. The combined treatment approach not only significantly inhibited the growth of drug-resistant xenografts, but also significantly influenced tissue morphology, inhibited the proliferation of drug-resistant transplanted tumors, and promoted their apoptosis. H&E staining showed that the tumor cell necrosis in the combination group was more pronounced. Additionally, H&E staining of the main organs, including the heart, liver, spleen, lung and kidney, showed no obvious lesions, suggesting low systemic toxicity of AP-7/DDP co-administration (Supplementary Figure S6). These results illustrate that the combination treatment with AP-7 and DDP enhances the mouse tumor necrosis, and its curative effect is better than that of AP-7 or DDP alone.

## 4 Discussion

Currently, multidrug resistance is a common occurrence in the clinical treatment of NSCLC (Watanabe et al., 2017). Although chemotherapy remains the primary approach for NSCLC treatment by inhibiting tumor proliferation and metastasis (Pirker, 2020), the development of multidrug resistance due to the DNA damage response of tumor cells limits the effectiveness of first-line chemotherapy drugs like DDP (Galluzzi et al., 2012; Kiss et al., 2021). Additionally, the eventual acquisition of resistance to new first-line standard EGFR-TKIs in EGFR-mutant NSCLC is almost unavoidable (Song et al., 2021). The mechanisms underlying EGFR-TKI resistance are complex and not fully understood, involving EGFR mutations, MET amplification, and overexpression of hepatocyte growth factor (HGF). Therefore, it is crucial and necessary to elucidate the key determinants of multidrug resistance to chemotherapy and targeted therapies in NSCLC. In our study, we investigated the DNA damage response in multidrug-resistant NSCLC cells following treatment with chemotherapy drugs and/or EGFR-TKIs. We observed DNA damage repair in multidrug-resistant NSCLC cells after 72 h of treatment with these agents. Our findings suggest that DNA damage repair plays a significant role in the resistance of multidrug-resistant NSCLC cells to chemotherapy and EGFR-TKIs.

Mechanistically, the treatment of drugs induces DNA double-strand breaks in tumor cells, causing the activation of ATM and ATR. This activation leads to the phosphorylation of Chk-1, which in turn triggers downstream activation of p53-associated DNA damage checkpoint and cell-cycle arrest. This cell-cycle arrest provides time for DNA repair and inhibits apoptosis (Zhao and Piwnica-Worms, 2001; Caeser and Sen, 2020). In our study, we observed that DDP treatment activated the Chk1/CDK1 DNA damage response pathway and induced cell cycle arrest at various stages in three multidrug-resistant NSCLC cell lines. This suggests that inhibiting the Chk1/CDK1 DNA damage response pathway may reduce DNA damage repair and enhance the cytotoxicity of DDP. Previous research by Bourgeois et al. demonstrated that inhibition of Chk1 by MK-8776 resulted in downregulation of the DNA repair protein RAD51 and severe DNA damage in pulmonary arterial hypertension (PAH) (Bourgeois et al., 2019). However, it is worth noting that there are currently relatively few drugs available that specifically target the DNA damage response pathway in drug-resistant cancers. Further extensive studies are needed to fully understand the potential of these drugs in the treatment of multidrug-resistant NSCLC (Babiker et al., 2017; Cui et al., 2019). Our aim is to develop a novel strategy to improve treatment outcomes and overcome multidrug resistance in NSCLC by targeting DNA damage-related pathways.

The marine environment is recognized as a rich source of numerous bioactive compounds that have been utilized as therapeutic agents in clinical settings, such as Cytarabine, Trabectedin, and Panobinostat (Hao et al., 2019; Jiao et al., 2021). The discovery of marine compounds with potential antitumor properties offers a novel approach to cancer treatment. Among marine-derived metabolites, aapatamine has been evaluated for its multiple anti-cancer activities (Gong et al., 2020b), including inhibiting growth of osteosarcoma cells and chronic myeloid leukaemia (CML) cells by arresting cell cycle (Jin et al., 2011b), and repressing tumorigenesis and progression of NSCLC by preventing cell cycle regulation drivers and the upstream regulator cascades (Gong et al., 2020b). Nevertheless, the anti-cancer effect and the underlying mechanisms of aaptamine in multidrug-resistant NSCLC are still not reported. In our study, we have identified the therapeutic potential of AP-7, a marine sponge-derived alkaloid in multidrug-resistant NSCLC cells. Our research has demonstrated that AP-7 inhibits the activation of Chk1, thereby reducing DNA damage repair through the blockade of the Chk1/CDK1 related DNA damage response pathway. Additionally, AP-7 has been shown to induce tumor cell apoptosis in multidrug-resistant NSCLC cells. Furthermore, our findings indicate that AP-7 effectively restores the sensitivity of multidrug-resistant NSCLC cells to DDP both *in vitro* and *in vivo* by targeting the DNA damage response pathway. These results highlight the promising role of AP-7 as a potential therapeutic agent for overcoming multidrug resistance in NSCLC.

## 5 Conclusion

Our findings indicate that sponge-derived alkaloid AP-7 effectively inhibits the activation of Chk1-related DNA damage response pathway induced by drug therapy. This inhibition prevents DNA damage repair and subsequently inhibits the proliferation of multidrug-resistant NSCLC cells, leading to increased tumor cell apoptosis. These results suggest that AP-7 may have the potential to enhance the sensitivity of multidrug-resistant NSCLC cells to DDP, making it a potential sensitizer in the treatment of this type of cancer.

## Data Availability

The original contributions presented in the study are included in the article/Supplementary Material, further inquiries can be directed to the corresponding authors.
