# Supplementary material for: Sponge-derived alkaloid AP-7 as a sensitizer to cisplatin in the treatment of multidrug-resistant NSCLC via Chk1-dependent mechanisms
Source: Front Pharmacol. 2024 Jul 9;15:1423684. doi: 10.3389/fphar.2024.1423684 (PMC11263074; doi:10.3389/fphar.2024.1423684)
Supplement: Supplementary file 1 [file DataSheet1.docx]

**Supporting Information for Publication**

**Title：**

Sponge-derived alkaloid AP-7 as a sensitizer to cisplatin in the treatment of multidrug-resistant NSCLC via Chk1-dependent mechanisms

**Author information**

Li Guan^1^, Ya-hui Liao^1^, Meng-xue Cao, Li-yun Liu, Hai-tao Xue, Hong-rui Zhu, Chang-hao Bian, Fan Yang, Hou-wen Lin*, Hong-ze Liao*, Fan Sun*

Li Guan and Ya-hui Liao contributed equally to this work.

**Corresponding Authors**

**Fan Sun**-Research Center for Marine Drugs, State Key Laboratory of Oncogenes and Related Genes, Department of Pharmacy, Renji Hospital, School of Medicine, Shanghai JiaoTong University, Shanghai 200127, People’s Republic of China; Email: sunfan@renji.com

**Hong-ze Liao**-Research Center for Marine Drugs, State Key Laboratory of Oncogenes and Related Genes, Department of Pharmacy, Renji Hospital, School of Medicine, Shanghai JiaoTong University, Shanghai 200127, People’s Republic of China; Email: liao0055@e.ntu.edu.sg

**Hou-wen Lin**-Research Center for Marine Drugs, State Key Laboratory of Oncogenes and Related Genes, Department of Pharmacy, Renji Hospital, School of Medicine, Shanghai JiaoTong University, Shanghai 200127, People’s Republic of China; Email: linhouwen@renji.com

**Table 1. The IC_50_ values and resistance-index (RI) of cisplatin, gemcitabine, vinorelbine, pemetrexed and 5-fluorouracil on A549 and A549/DDP cells.**


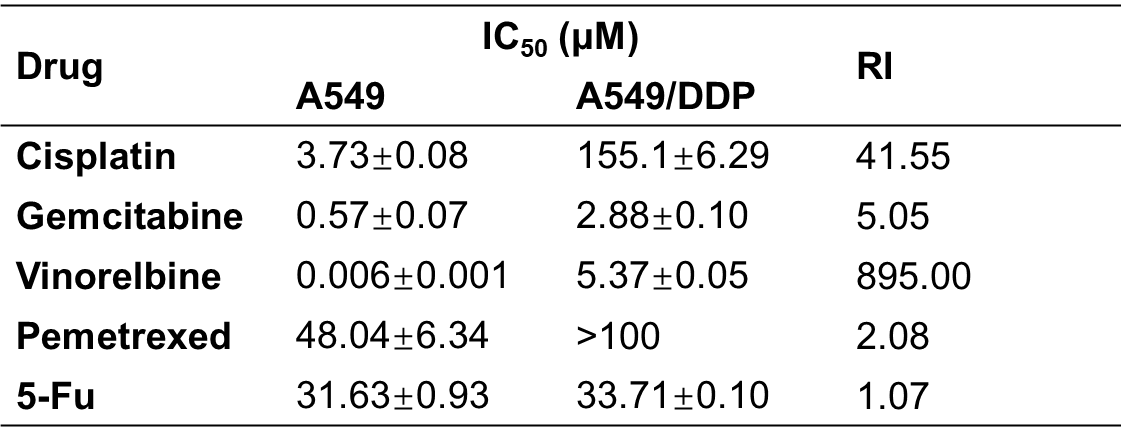


**Table 2. The IC_50_ values and RI of gefitinib, cisplatin and gemcitabine on PC9 and PC9-GR cells.**


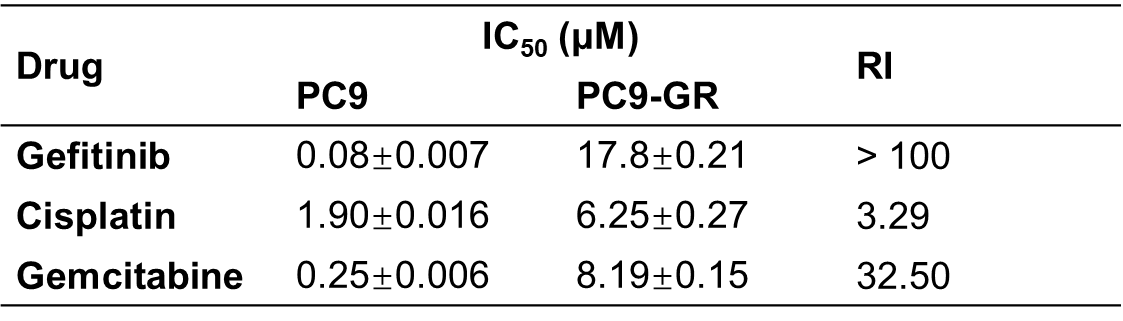


**Table 3. The IC_50_ values and RI of cisplatin and gemcitabine on PC9-NC and PC9-Nanog^+^ cells.**


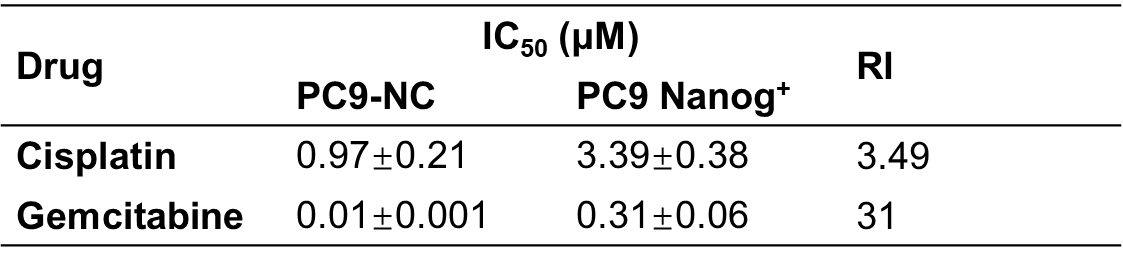


**Table 4. The IC_50_ values of AP-7 on PC9-GR, A549/DDP and PC9-Nanog^+^ cells.**


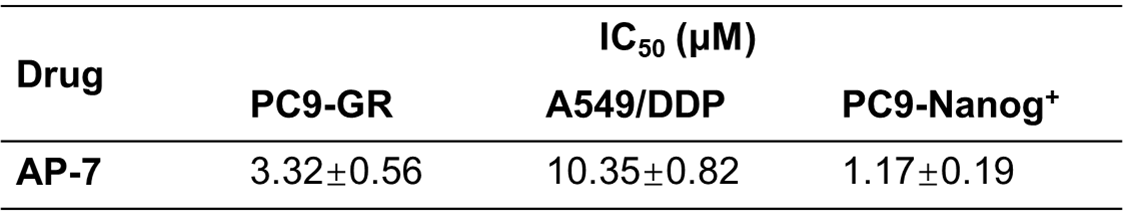


**Table 5. T/C ratio of different treatment groups. T/C% = TRTV/CRTV × 100%; TRTV, relative tumor volume after drug treatment; CRTV, relative tumor volume of vehicle control.**

**
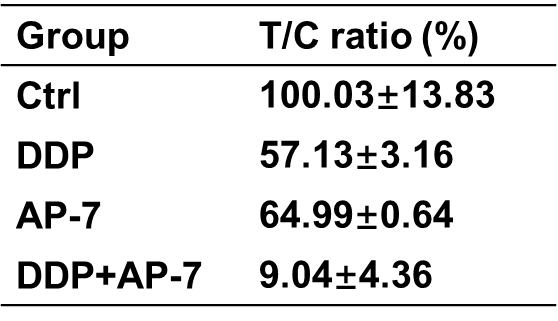
**

**Table 6. Primary antibodies used in the study.**


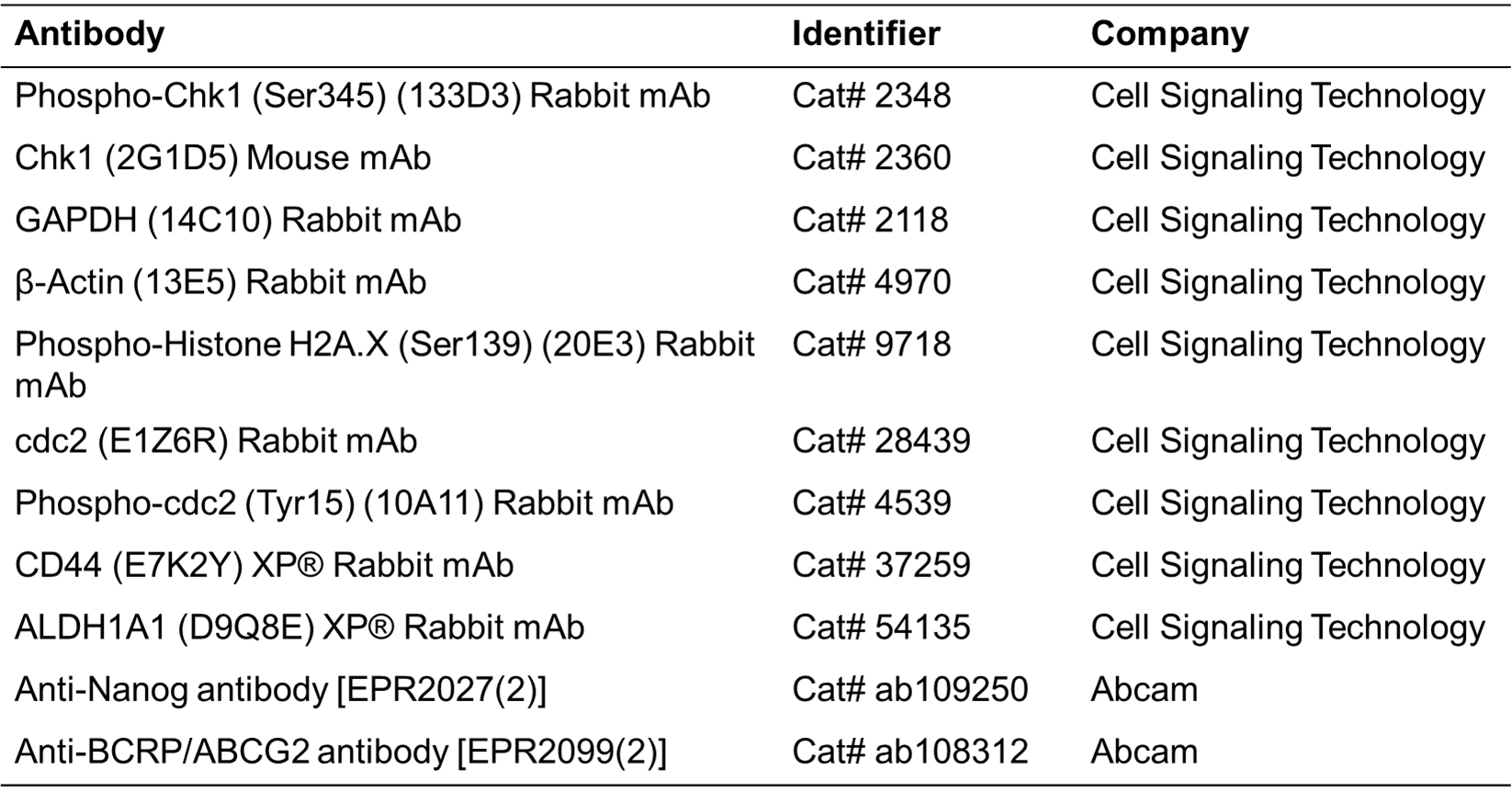


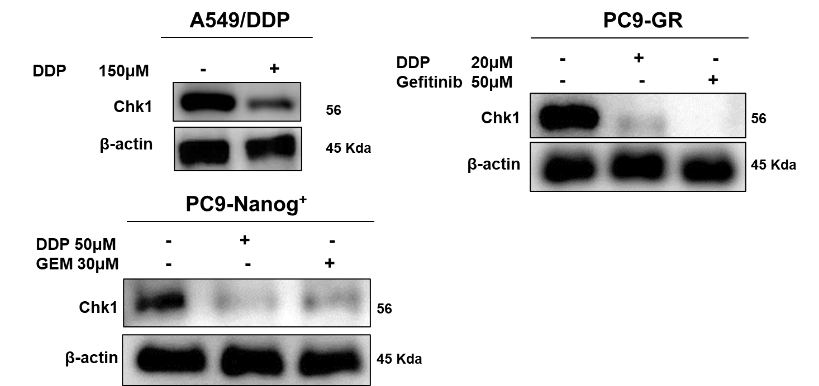


**Fig. S1 The expression levels of Chk1 in A549/DDP, PC9-GR, and PC9-Nanog^+^ cells after treatment with chemotherapy drugs and EGFR-TKIs.**

**
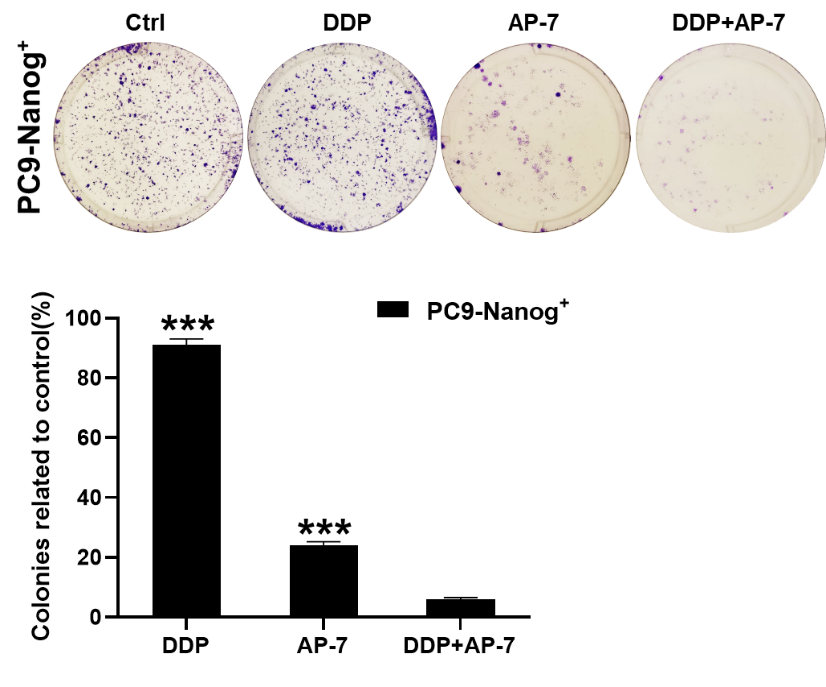
**

**Fig. S2 AP-7 inhibited the formation of PC9-Nanog^+^ cells colonies ***p<0.001, compared with DDP+AP-7 group.**

**
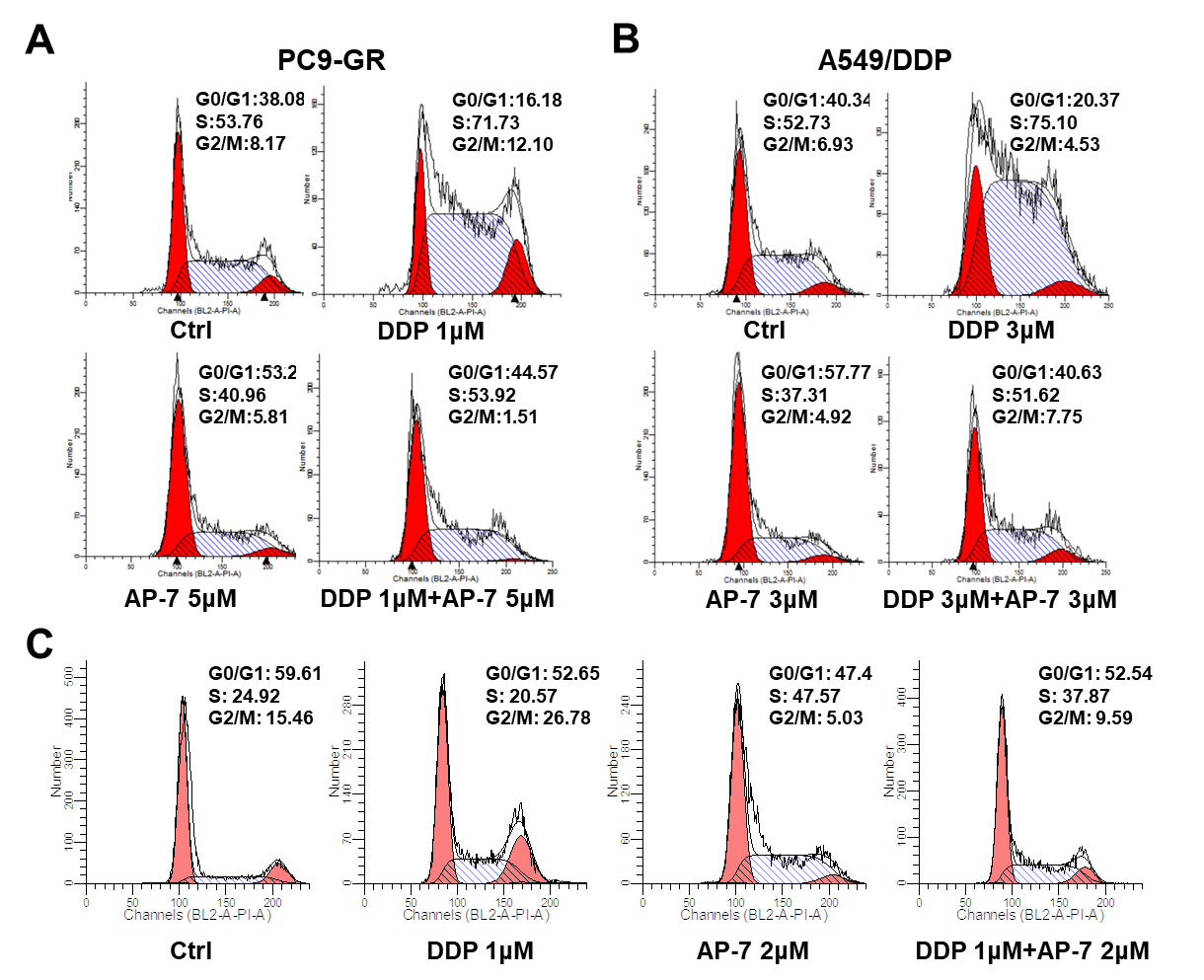
**

**Fig. S3 Cell cycle distribution of (A) PC9-GR, (B) A549/DDP and (C) PC9-Nanog^+^ cells after treatment with AP-7 and DDP was tested by flow cytometric assay.**


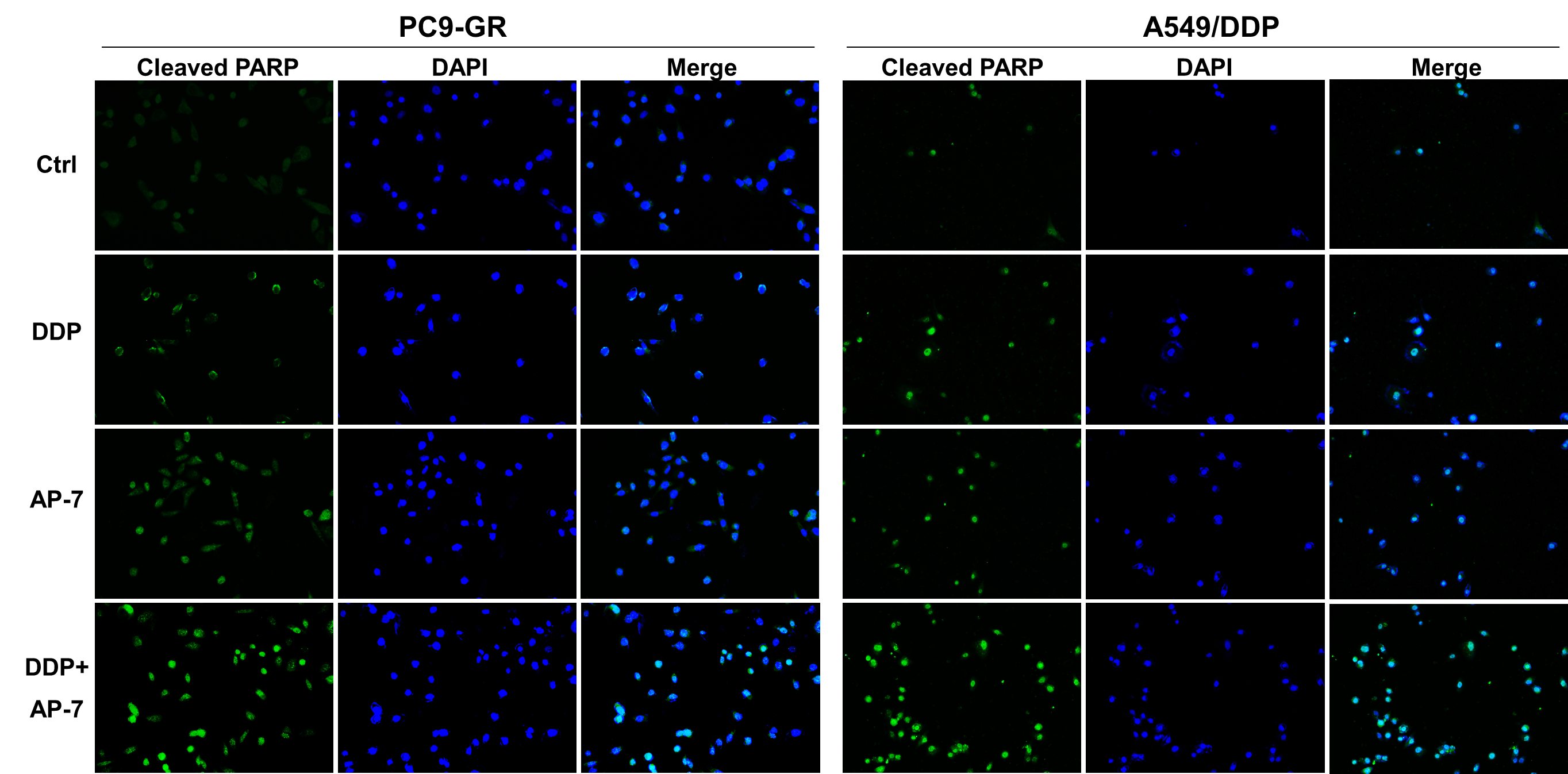


**Fig. S4 PC9-GR and A549/DDP were treated with DDP and AP-7 for 72 hours, respectively, and the apoptosis cells was determined by Cleaved PARP staining.**


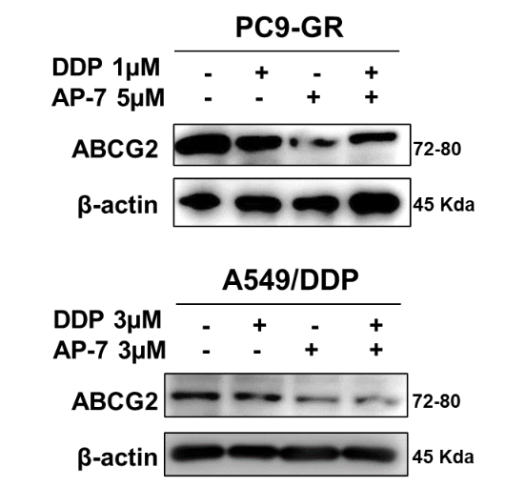


**Fig. S5 The expression levels of ABCG2 in PC9-GR and A549/DDP cells after treated with AP-7 and DDP for 24 hours.**

**
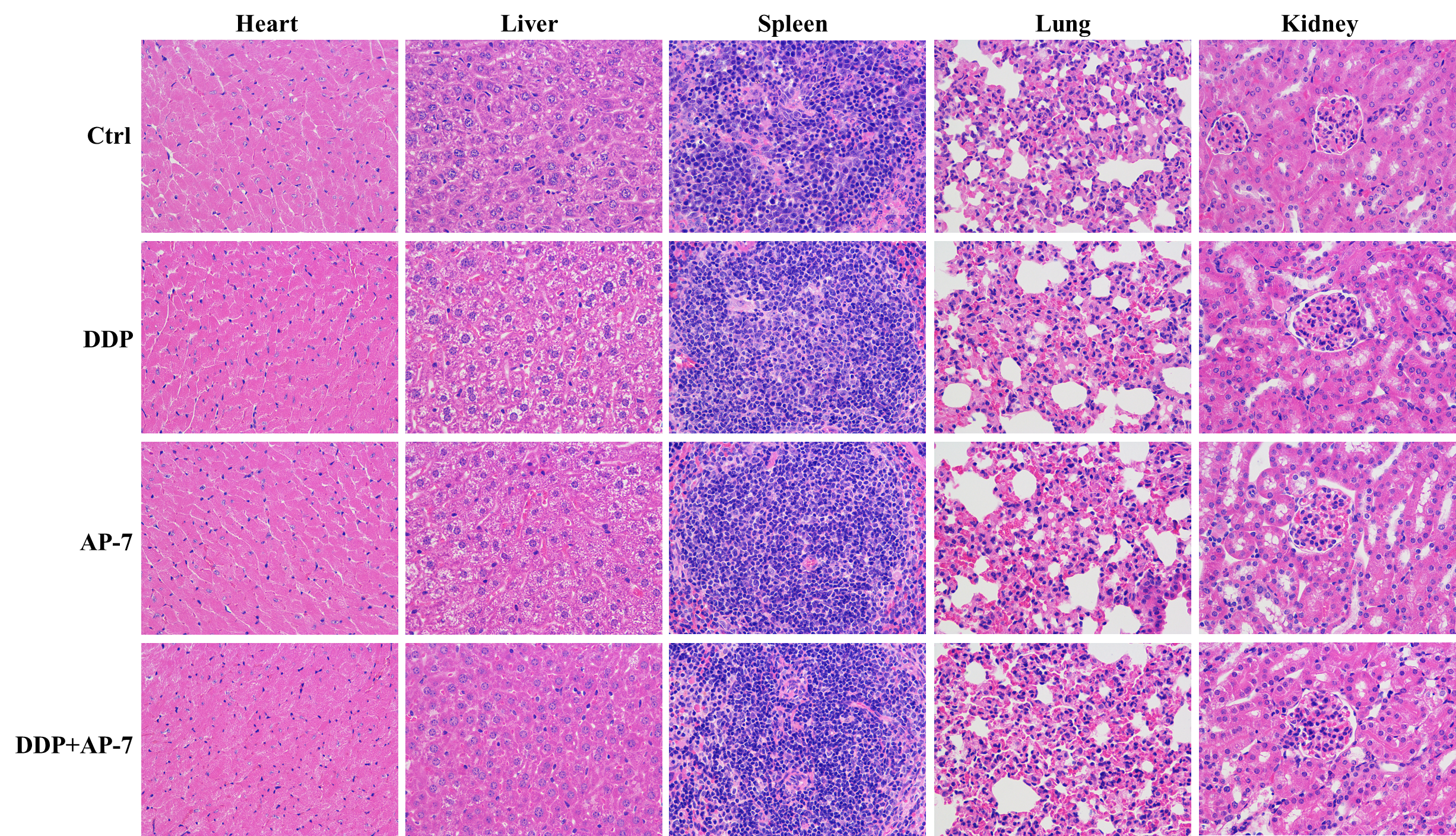
**

**Fig. S6 Preliminary safety assessment. H&E staining of major organs in different treatment groups (H&E 400×).**
